# A comparative analysis of the doctoral regulations at the medical faculties in Germany

**DOI:** 10.7150/ijms.92167

**Published:** 2024-02-12

**Authors:** Sarah Altenberger, Roman Leischik, Richard Vollenberg, Jan Peter Ehlers, Markus Strauss

**Affiliations:** 1 Department Didactics and Educational Research in Health Science, Faculty of Health, University of Witten/Herdecke, 58455 Witten, Germany.; 2 Department of Cardiology, Faculty of Health, School of Medicine, University of Witten/Herdecke, 58455 Witten, Germany.; 3 Department of Medicine B, Gastroenterology and Hepatology, University Hospital Muenster, 48149 Muenster, Germany.; 4 Department of Cardiology I—Coronary and Peripheral Vascular Disease, Heart Failure Medicine, University Hospital Muenster, Cardiol, 48149 Muenster, Germany.

**Keywords:** doctorate, doctoral thesis, doctoral regulations, equal opportunities, medical education

## Abstract

**Objective:** In order to be allowed to use the title “Dr. med.” in Germany, an independent scientific achievement under the supervision of an established scientist is necessary. The research question, analysis and results are essentially carried out and developed independently by the doctoral student. The doctorate serves as proof that the doctoral candidate is capable of independent academic work. The acquisition of scientific skills and knowledge is of particular importance in medicine, as Germany´s international competitiveness is based on the education of today´s young academics. Fair conditions and uniform quality standards for doctoral studies are therefore indispensable to attract future young scientists at an early stage.

**Methods:** The currently valid doctoral regulations of the medical faculties in Germany were analysed with regards to the following target criteria; update date, dissertation language, possibility of publication-based dissertation and its details (number of first and total authorships, publication organ), knowledge of methods and consideration of "Good Medical Practice" (GMP), plagiarism check, review process and disputation.

**Results:** All faculties with the right to award doctorates, and, thus 40 valid regulations were included in the analysis. This revealed a great divergence in the requirements for doctoral candidates. Although a publication-based doctorate is now possible at 93% (n=37) of the faculties, in addition to the monographic dissertation, the required first and total authorships vary from one required first authorship (n=26, 70%) to two or three first authorships (n=5, 14%), as well as some faculties having no information regarding the number of publications (n=6, 16%). The quality of the publication organ was not described in detail in seven faculties (19%). To ensure quality, requirements have increasingly been anchored in the regulations, so that 22 regulations (56%) now stipulate participation in courses on GMP or qualification programmes. The regulations leave a lot of room for manoeuvre in terms of content and do not allow for comparability of the conditions for preparing doctoral researchers. The specifications range from mere mention, to instruction, to compulsory course participation. Another means of quality assurance is the prevention of plagiarism through the applications of software systems. However, this simple and effective means is not yet mentioned in 65% of the regulations (n=26). While the other regulations make use of this possibility, it is not an obligatory application. A total of 34 regulations provide for the regular drawing up of a supervision agreement to define the rights and obligations of the actors involved.

**Conclusion:** The analysis showed a divergent picture. Although imprecise regulations or gaps in information allow scope for design, they also prevent transparency. Despite revisions of many regulations in the past, these revisions have not led to any significant harmonisation. The implementation of standardised and structured doctoral programmes is desirable and could be tackled within the framework of the planned amendment of medical studies. This opens up the possibility of dealing efficiently with the scarce resource of time in the face of competing curriculum content and of making a doctoral project more attractive to potential young scientists at an early stage.

## Introduction

In Germany, the practice of medicine requires a state-issued licence to practice medicine 1. This can be applied for after successfully completing a course of study of six years and three months. The academic title of Dr. med. can only be obtained in Germany by working on a scientific topic. Most of these doctoral projects start in parallel to the study of human medicine 2, 3. While the focus is on German medical faculties, it's important to acknowledge that medical education structures and doctoral requirements can vary globally. These differences in medical education content and structures pose a challenge when comparing across countries.

The medical doctoral thesis is a piece of scientific work produced independently by the doctoral candidate, which is intended to demonstrate their ability to think and work scientifically 4. Doctoral performance consists of the writing of a dissertation, which can be of an experimental, clinical, statistical or theoretical nature, and successful disputation. In addition to a monographic dissertation, a publication-based (cumulative) doctorate is now also possible at many faculties. This is characterised by the fact that the written dissertation performance is usually achieved through thematically related publications that have been published in peer-reviewed journals. The requirements for the doctorate are defined by the faculties in their respective doctoral regulations. The doctoral thesis is supervised by a supervisor. A person who may hold this role is regulated by the doctoral regulations. As a rule, these are habilitated university teachers or professors.

About 60% of the doctors working in Germany hold a doctorate, and medicine has the highest number of doctorates compared to other courses of study 5-7. The development of scientific competences is of particular importance in medicine, as medical knowledge is constantly changing and the basis of medical action and counselling should always be based on the latest scientific findings, in the sense of evidence-based medicine 8.

In addition, research projects are the engine of continuous further development in medicine. Scientificity is therefore a core medical competence that should be given appropriate priority throughout the entire period of training and continuing education 9. Transparent and uniform requirements for medical doctoral theses provide the basis for promoting young scientists and ensure international comparability.

The aim of this manuscript is to compare the requirements for the medical doctoral thesis on the basis of the valid doctoral regulations at German medical faculties, regarding criteria such as the assessment process, publication-based promotion, aspects of disputation and other factors. Based on this comparison, similarities and differences between the regulations will be highlighted to identify potential opportunities for optimizing the current status quo.

## Methods

### Study Design

The valid doctoral regulations of 40 medical faculties in Germany as of 12.06.2023 were included. The respective doctoral regulations were accessed through the official documents available on the faculties' websites.

### Inclusion Criteria

All doctoral regulations of state-accredited medical degree programmes in Germany that lead to the award of a licence to practice medicine issued in Germany were included as of 12 June 2023. The period of the included doctoral regulations extends from 2010 to 2023.

### Exclusion Criteria

Cooperation projects between German hospitals and foreign medical faculties with attainment of a foreign degree and universities and higher education institutions without the right to award doctorates or that lack the accreditation of a medical degree programme were not included in the analysis.

### Target Criteria

The doctoral regulations were analysed for the following target criteria: date of last update, dissertation language, possibility of publication-based promotion and its details (number of publications, number of first authorships, quality of publication organ), consideration of "Good Medical Practice" and methodological skills, plagiarism check, details of the review process, details of the disputation, overall grade.

### Goal of Analysis

The aim of this work is to examine the doctoral regulations of German accredited medical faculties on the basis of the above-mentioned target criteria.

### Statistical Analysis

The target criteria of dissertation language, requirements for the publication-based doctorate (number of first and total authorships, quality of the publication organ), plagiarism check, criteria of the disputation, methodological skills and GMP are given in absolute and relative frequencies. The date of the last update is described by maximum (max), minimum (min), median (quartile 2), quartile 1 (Q1) and quartile 3 (Q3).

## Results

**Figure 2:**
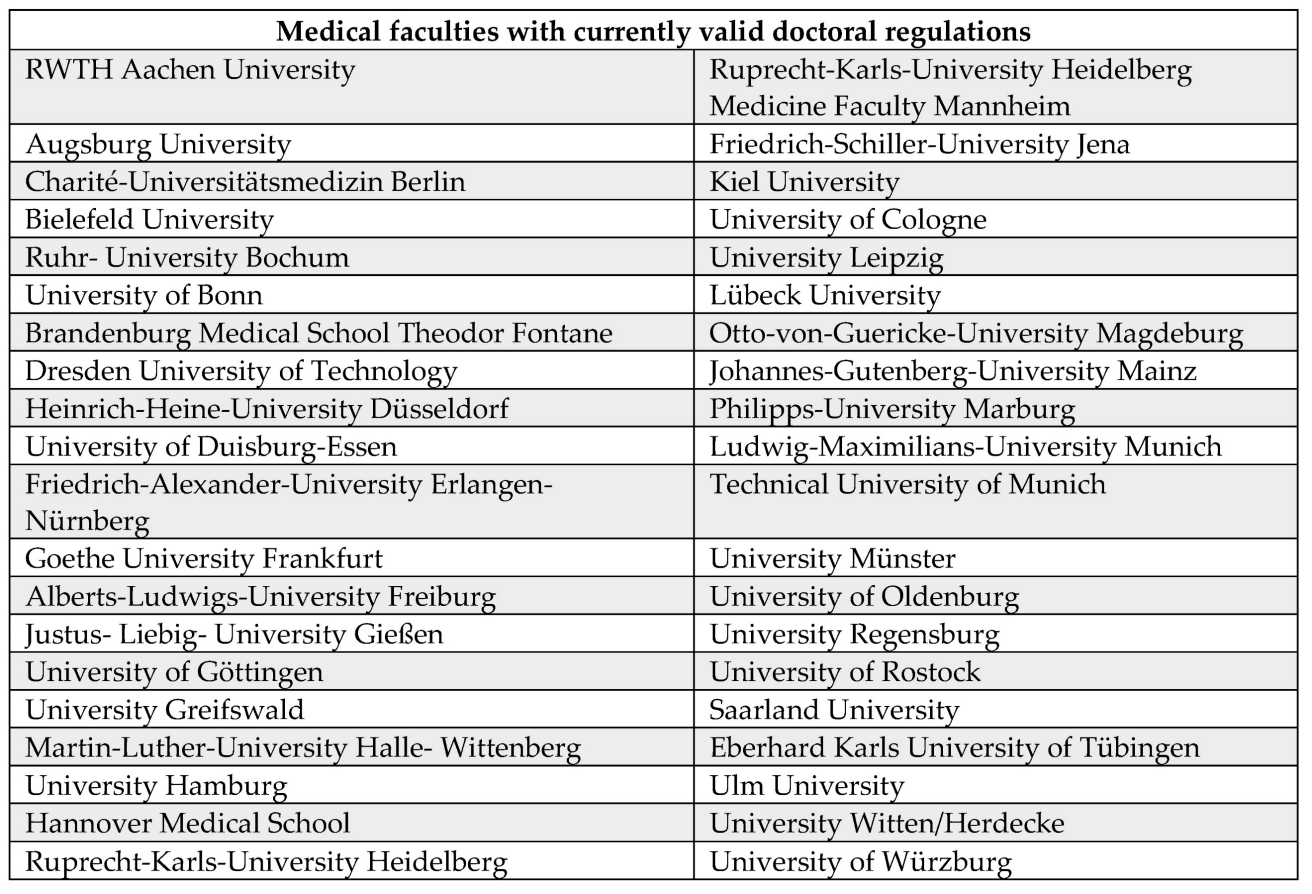
Universities with currently valid doctoral regulations.

Of a total of 44 medical faculties in Germany, 40 faculties have the right to award doctorates with web-based doctoral regulations (91%) and could be included in the analysis. Medical School Berlin, Medical School Hamburg, Health and Medical University Potsdam and Health and Medical University Erfurt (n=4, 9%) do not currently have the right to award doctorates in Germany and therefore do not have doctoral regulations. The medical faculties with valid doctoral regulations are listed in Figure 2.

### Age of the doctoral regulations

The timeliness of the doctoral regulations shows great divergence. On average, the time of the last revision was 4.2 years ago (Q1= 2, median=3, Q3=6). The most recent revision was less than a year ago, whereas the oldest revision was 13 years ago.

### Basic Requirements

A doctoral project is based on the doctoral regulations of the corresponding medical faculty. A specific date for the doctorate is not prescribed. A doctoral thesis can be started during the study of human medicine (usually possible after completion of the first state examination), but the process can only be completed after obtaining the licence to practice medicine. The rights and obligations of the doctoral candidate and supervisor can be laid down in a doctoral or supervision agreement. This serves to secure and specify the duties of the doctoral candidate and is obligatory in 85% (n=34) of the doctoral regulations examined.

Some universities (n=13, 33%) require that at least two "compulsory semesters" of study be completed at the respective university or that scientific work be carried out in the area of the medical faculty if the doctorate is to begin before completion of the degree programme 10, 11. If a doctoral project only takes place after completion of the medical studies, the doctoral candidate must re-enrol at the relevant university until the end of the procedure.

### Language

The universities specify in their regulations in which language the dissertation must be written. A total of 34 universities (85%) allow the dissertation to be written in either German or English. The rest (15%) do not give any specific information on this issue.

### Knowledge of methods

**Figure 4:**
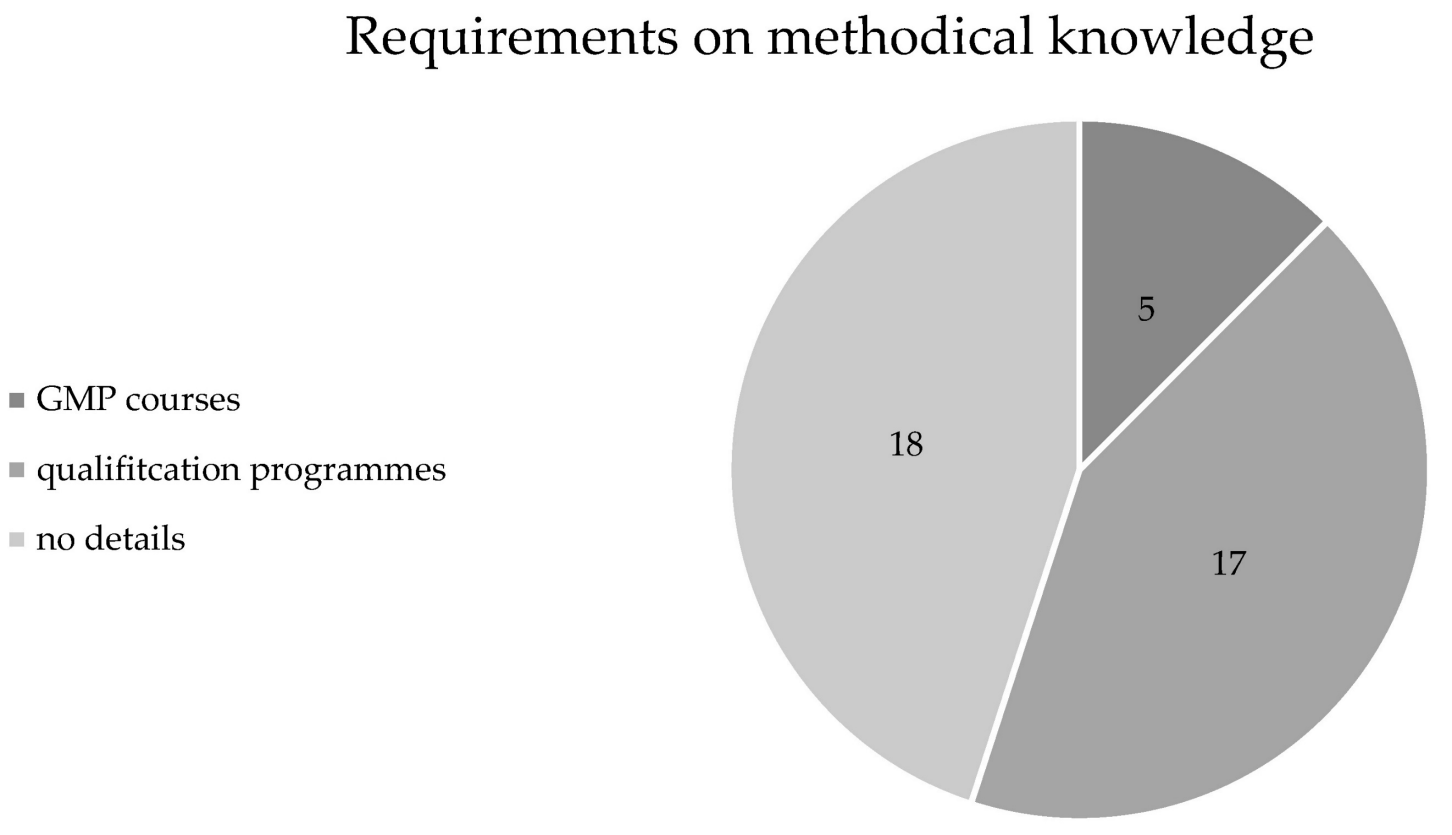
Requirements for methodical knowledge.

The basis for completing a doctorate is knowledge of the methodology of scientific work and research. The requirements for qualification programmes are varied in the regulations and are shown in Figure 4. In addition to the obligation to sign a declaration on working according to GMP (n=10, 25%) 12, some faculties stipulate the completion of compulsory courses on GMP or obligatory participation in qualification and training programmes (n=21, 53%) 13, 14.

### Possibility of a publication-based dissertation

In addition to the monographic dissertation, 93% (n=37) of the medical faculties also offer publication-based cumulative doctorates. The details of the publication-based dissertation show differences in their elaborations in terms of the required number of first and total authorships. While 26 faculties (70%) require only one first authorship, 4 (11%) provide for at least two first authorships and 1 (3%) requires three first authorships. In 81% (n=29) of the cases, the quality of the publication organ, e.g., journals with a review process or impact factor, is also taken into account.

Regarding the total number of authorships (including first authorship), 49% (n=18) of the universities require a total of one authorship. In addition, 35% (n=13) provide for two to three total authorships. Three universities each either make imprecise statements (n=3, 8%), such as "several", or do not explicitly comment on this point (n=3, 8%).

### Review process

The review of the dissertation is obligatory and decisive for the grading of the written part of the doctoral thesis. A distinction is made between internal and external reviewers, whereby external reviewers belong to another institution or foreign university. The internal review process is not specified in more detail in one faculty (3%). A total of 90% (n=36) of the regulations require two internal reviews. The Martin Luther University of Halle-Wittenberg additionally states that the internal reviewer cannot be the supervisor of the doctorate 15. At three medical faculties (8%), only one internal review is required, but in these cases an external review is obligatory. An obligatory external review is provided for in 45% (n=18) of the doctoral regulations. At 10 medical faculties (25%), an additional external review is required as soon as the internal review has resulted in the grade "summa cum laude" 16.

### Plagiarism check

Correct citation in a dissertation is essential to ensure the quality and protection of scientific work. In the meantime, universities have software systems at their disposal to check a dissertation for plagiarism. A total of 35% of universities provide information in this regard, such as the Hannover Medical School, where doctoral candidates are required to sign a declaration of consent for potential preventive plagiarism checks on their dissertations 13, 17. The majority (n=26, 65%) do not mention this topic in their doctoral regulations.

### Disputation

**Figure 5:**
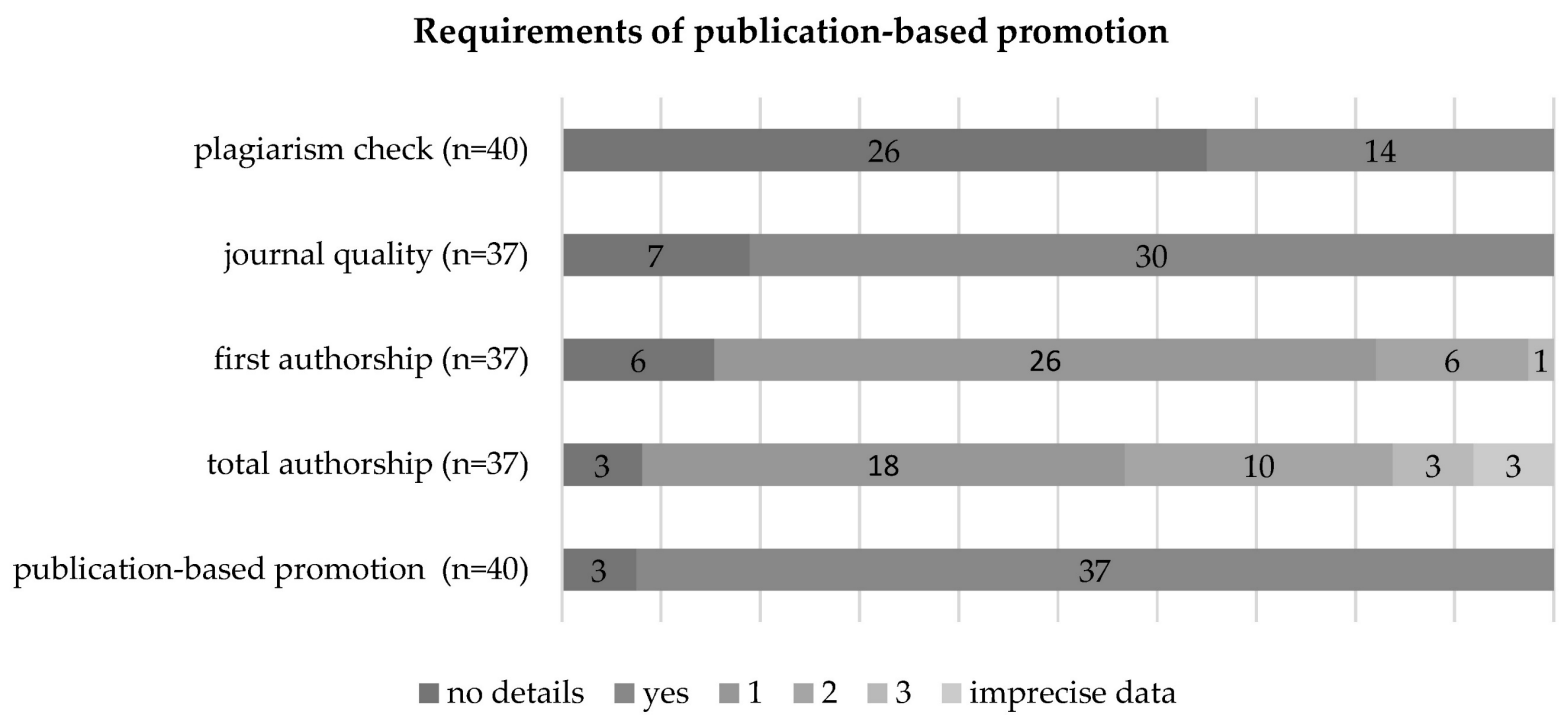
Requirements of publication-based promotion.

The disputation, or defence of the doctoral thesis, represents the second part of the doctorate, in which the broad knowledge of the scientific topic is to be presented and the results of the thesis can be discussed. A total of 73% (n=29) of the regulations provide for a public plenum, with 28% (n=11) restricting it to a defined group of people. The University of Bielefeld, for example, requires an intra-faculty defence 18, whereas the University of Bonn specifies a closed circle of people consisting of the first examiner, an examiner from a chosen discipline named by the applicant, which is outside the dissertation topic, and a doctoral adviser 19. Some universities make a distinction as soon as the predicate summa cum laude (s.c.l.) and magna cum laude (m.c.l.) are involved, so that from this point onwards the disputation has to be held in public 20. Regarding the examination area of the disputation, 33% (n=13) of the faculties include related research areas, whereas at 17 universities (43%) the defence focuses on the dissertation topic or its research areas. A total of 25% (n=10) of the faculties do not provide any information on the content of the oral performance (Figure 5).

### Grading and Weighting

The disputation is included in the overall grade at 37 medical faculties (93%). In most cases, the oral and written marks are weighted in a ratio of 1:3 21 or the marks are listed separately on the doctoral certificate 22. A special situation exists at the University of Tübingen, where the disputation is only assessed as passed or failed and a grade is given only for the dissertation thesis 13.

## Discussion

The international comparison of academic career paths is difficult due to the lack of uniform structures. The Bologna Process of 1999 for unifying the higher education systems of European countries has led to adjustments, but there is still a great deal of divergence with regards to medical doctorates 23.

### Exploring M.D./Ph.D. Programs: Opportunities, Challenges, and Career Trajectories

For example, Austria and the U.S. award the titles Dr. med. univ. or M.D. ("Doctor of Medicine"), respectively, in the sense of a professional doctorate 24. This may be followed by an additional doctoral programme or a Ph.D. programme leading to the title of Dr. med. scient. or Ph.D., respectively 25, 26. Ph.D. stands for "Doctor of Philosophy" and is not subject-specific in its naming. Obtaining the title thus requires an individual research achievement in the form of a dissertation and disputation. In addition, there is another possibility for students who want to dedicate themselves to research at an early stage through the so-called M.D./Ph.D. programmes, which were introduced in the U.S. in the middle of the last century 26, 27. Within the framework of medical scientists programmes (MSTP), two degree programmes are combined so that the medical degree (M.D.) and the research degree (Ph.D.) can be obtained simultaneously over a period of 7-9 years 28.

Although the U.S. has historically faced challenges in recruiting enough young scholars 29, 30, M.D./Ph.D. programmes are only accessible to a small proportion of students 27. On the one hand, they require a high commitment to work, so that they are accessible primarily to highly motivated students 31, and, on the other hand, the programmes are associated with high costs for the faculty and National Institutes of Health (NIH) through the waiving of tuition fees or the awarding of scholarships 27. Therefore, the M.D./Ph.D. programmes seem to make sense for students who want to prepare for a career in science at an early stage and ensure secure career paths for graduates afterwards 28, 32. Nevertheless, there is a declining number of graduates, and attrition rates from MD/PhD programs are reported to range between 10% and 28.5% 33, 34. The main reasons against such programmes seem to be, among others, the lengthening of the training period, the higher costs due to an extended training period, the high competition for professorships and the compatibility of family and career 35. However, these statements cannot be generalized. Beyond the challenges inherent in these programs, numerous advantages are apparent. These manifest in the graduates' high-caliber research endeavors and the ensuing prospects for academic career advancement 28.

### European Adaptations of Ph.D. Programs in Medicine: Structure, Challenges, and Career Implications

Programmes based on the American Ph.D. programme have also been established at European universities. Among them are German universities, exemplified by the Ludwig Maximilian University of Munich, where the Ph.D. can be achieved through a structured three-year, full-time doctoral programme after completing medical studies 36. There is also a Clinical Scientist Programme at University Witten/Herdecke, which, parallel to residency training, provides access to experimental research through the development of a biomedical project and can be completed with a Ph.D. 37. Since October 2020, the private Paracelsus Medical University in Salzburg has also been offering a Ph.D. programme in addition to the human medicine programme, through which the internationally recognised title of Ph.D. can be obtained within three years 38. In Switzerland, a study by the University of Geneva showed that after the introduction of M.D./Ph.D. programmes, various obstacles exist in the form of the double burden of clinical work and allocated research time, insufficient mentoring and funding and a difficult work-life balance 39. Certainly, one challenge here is that, in contrast to the U.S., in Germany, Austria or Switzerland Ph.D. programmes are completed after medical school. Although scholarships and funding programmes are available, the long-term goal of clinical work delays the start of the career and residency training accordingly. Nevertheless, as in the U.S., there seems to be an advantage for the further professional career of graduates, especially if they wish to pursue an academic career path 28, 32, 39.

### Expanding Scientific Competency Training in German Medical Studies: A Call for Standardization and Enhancement

The teaching of scientific skills is a fundamental component of medical studies in Germany. According to the currently valid medical licensing regulations, the goal of a doctor trained "scientifically and practically in medicine" is mentioned, although the framework conditions for this scientific competence training are not further specified 1. The regulations merely state that "the scientific and methodical knowledge" is to be imparted by the teacher within the lectures 1. The Medical Faculty Association (Medizinische Fakultätentag) and the German Science Council (Deutscher Wissenschaftsrat) see a need to further expand this transfer of competencies, to standardise it and to make it more visible 9, 40, 41. Ultimately, the early teaching of scientific skills and the introduction to research activities not only serve to ensure evidence-based and high-quality medical care by the trained physicians but also to recruit young scientists 9. However, the compulsory curriculum leaves little room for intensive engagement with the field of research 9. Medical doctorates therefore play an important role as a building block, and despite the decline in the number of completed doctorates, they still represent an important building block for many students in their own career planning 42-44. In this context, it must be mentioned that the design of the conditions of the doctorate is the responsibility of the medical faculties.

### Challenges in Standardizing Medical Doctoral Regulations in Germany: A Quest for Uniformity and Quality Assurance

Uniform, nationally regulated requirements, as in the design of the compulsory curriculum of medical studies on the basis of the approbation regulation (Approbationsordnung), do not yet exist with regards to the medical doctorate. In the past, the work of Sorg et al. 4 came to the conclusion that the standardisation of doctoral regulations would be desirable. The ongoing criticism and controversial discussions about the quality of the medical doctorate also make this revision seem sensible 4, 45, 46. It should therefore be acknowledged that almost all doctoral regulations have undergone a revision since the last analysis by Sorg et al. 4. Over time, a process of standardization and objectification of individual criteria can be observed. Currently, 85% (n=34) of regulations mandate the conclusion of a doctoral agreement, whereas this was only considered an obligatory parameter in 70% (n=26) of doctoral regulations in the past. In the analysis by Sorg et al. 4, plagiarism checks in 2016 did not demonstrate consistent practice, and no medical faculty performed standardized checks on submitted works using plagiarism software. Presently, 35% (=14) of faculties report regularly using such software. Furthermore, nowadays, publication-based doctoral programs are considered mandatory in 93% (n=37) of regulations, compared to a mention in 70% (n=26) of doctoral regulations in 2016. Additionally, in recent years, there has been a significant objectification of the review process through the obligatory requirement to name at least one external reviewer. Currently, 45% (n=18) of regulations mandate the submission of an external assessment in the review process, whereas in 2016, it was stipulated as an obligatory criterion in only 16% (n=6) of regulations.

Unfortunately, although these updates have led to more detailed requirements in some places, they have contributed little to standardisation. Instead, the analysis carried out here continues to show a picture of divergent regulations and specifications which, in part due to imprecise formulations, allow leeway for design but contribute less to transparency, equal opportunities and the assurance of quality standards. In this way, the doctorate follows the example of other academic qualification processes in the sense of a habilitation or adjunct professorship (außerplanmäßige Professur or apl. Professur), as there is also evidence of great divergence with regards to the regulations 47, 48.

Approximately 60 % of doctors working in Germany hold a doctorate, and medicine has the highest number of doctorates compared to other courses of study 5-7. Most of these doctoral projects are started during studies, but a not irrelevant proportion do not lead to the successful completion of the doctorate 2, 3. Inadequate mentoring, in particular, has a negative influence on the chances of success of the doctoral project 3, 49. It is therefore to be welcomed that a mentoring agreement to mutually secure and regulate the rights and obligations of doctoral researchers and mentors is now firmly anchored as a requirement in 34 doctoral regulations (85%). In 2014, only 26 faculties had such a supervision agreement 4. Another factor influencing the success of a doctoral project is the structured preparation for the doctorate 3, 49. Therefore, a mandatory, uniform and structured teaching of competencies would make sense and would also be in line with the National Competence-Based Catalogue of Learning Goals in Medicine (Nationalen Kompetenzbasierten Lernzielkatalog Medizin). This recommends that all students should master the scientific methodological basis 50. Due to the ongoing criticism of the medical dissertation 4, 45, 46, measures have increasingly been anchored in the regulations to ensure higher standards and more quality assurance. One possibility is compulsory or voluntary participation in doctoral preparation courses and a commitment to work according to the guidelines for ensuring "Good Medical Practice" (GMP). The latter are 19 guidelines developed by the German Research Foundation (Deutsche Forschungsgemeinschaft; DFG), the legally binding implementation of which is a prerequisite for receiving funding from the DFG 51.

It should therefore be emphasised that although mandatory participation in qualification programmes (n=17, 43%) or courses to ensure GMP (n=5, 13%) has increased since the work of Sorg et al. 4, 45% (n=18) of the faculties do not provide any information on methodological knowledge or GMP. In these cases, it remains open as to wether voluntary participation in qualification programmes is offered. In order to be able to stand up to international standards, an adaptation and obligation to teach the basics would be a desirable goal, along the lines of doctoral programmes or Ph.D. programmes, in which the teaching of competencies through course participation is mandatory.

With regards to the question of the scientific education of medical students in general, it is worthwhile to consider the current discussions surrounding the amendment of medical studies. In 2015, the then Federal Government reacted to the ongoing criticism of medical studies and formed a working group that was given the task of implementing the so-called Master Plan for Medical Studies 2020 (Masterplan Medizinstudium 2020). The Master Plan for Medical Studies 2020 aims to ensure a modern and practice-oriented education for medical students. One of its primary objectives is to promote a strong foundation in scientific knowledge. In particular, the plan addresses the issue of teaching scientific skills and provides for the "introduction of a certificate of achievement for the structured teaching of scientific skills" 52. This is to be integrated into the curricula as a scientific thesis and provided between the first and second section of the medical examination 53. The scientific exploration of a subject-specific topic is aimed at enabling them to develop fundamental skills in research methodology, including conducting experiments, analyzing data, and interpreting scientific literature. Furthermore, it helps students to grasp the significance of evidence-based medicine, thereby enhancing their ability to think critically and make informed decisions in clinical practice. Ultimately, they are empowered to actively contribute to scientific discourse and the advancement of medical knowledge.

An alternative to the current situation is the professional doctorate that has already been presented. Following the example of the Anglo-American region, Dr. med. univ. or M.D. could be awarded with the attainment of the licence to practice medicine 54, 55. The scientific performance for the professional doctorate would be achieved through the study programme itself and through the compulsory preparation of a scientific thesis that would be introduced in the future.

### Publication-based doctorate and review process

All in all, it remains to be said that the responsibility for shaping the requirements for a medical doctorate continues to lie with each individual medical faculty.

The publication-based doctorate is now an established form of writing at almost all medical faculties (93%) in Germany. However, the detailed design of the criteria for a publication-based doctorate remains divergent. In particular, there are clear differences in the required total number of authorships.

To ensure a uniform quality of the publications, specifications on the publication organ are desirable. These requirements (e.g., via the impact factor) are described in more detail in 30 (81%) of the doctoral regulations. In the case of publication-based dissertations, a more comprehensive scientific classification by means of an additional synopsis seems to make sense. In almost half (n=18, 49%) of the faculties, this would not come into play because only one publication is required, but, in the case of several publications, outlining a thematic connection and classification in the scientific context can be useful.

After the dissertation has been successfully completed, it is submitted and reviewed. This process is regulated differently at the faculties and can take place through the involvement of internal and/or external reviewers. In some cases the supervisor also acts as a reviewer, so questions regarding necessary independence and objectivity may be raised 45. Dissertations in Germany are traditionally graded using Latin honors. The highest achievable grade is equivalent to 'summa cum laude.' (s.c.l.). This grading signifies the highest level of praise, representing an exceptional performance deserving of special recognition. At some universities, a differentiation takes place as soon as the predicate summa cum laude has been awarded within the framework of the internal assessment. In this case, an additional external assessment should take place and is useful for maintaining and verifying objectivity in the highest grade awarded. In order to prevent a lack of objectivity, another possibility is the anonymous and external review, which has already been called for several times, based on the procedures for publications within the framework of a peer-review process 4, 45, 56, 57.

### Implementation of Plagiarism Software

In order to prevent the theft of intellectual property, 35% (n=14) of the faculties have now introduced the use of plagiarism software in their regulations. Yet, there's no mention in any regulation or documentation regarding the specific plagiarism detection software utilized by the individual faculty. As this is an easy-to-use tool that would significantly help the reputation and quality of medical dissertations, the widespread use of plagiarism software before the dissertation is submitted for review would be sensible and desirable. Nevertheless, it should be mentioned that this was only planned at six faculties in 2014 and that the number has at least doubled since then 4.

### Diversity in Disputations: Varied Formats and Participants in Doctoral Defenses

The final hurdle of the doctoral project, the disputation, also seems to have diversity among the regulations. Differences can be seen in the content and the group of people to whom the defence is open. A total of 73% (n=29) of the regulations provide for a public plenum, with 28% (n=11) restricting this to a defined group of people. The University of Bielefeld, for example, requires an internal faculty defence 18, whereas the University of Bonn specifies the closed circle of people consisting of the first examiner, an examiner from a subject area named by the applicant that lies outside the dissertation topic and a doctoral assessor 19. Similar to the assessment, some universities make a distinction as soon as the predicates s.c.l. and m.c.l. are concerned, so that from this point onwards the disputation must be held in public 20. The required content of the disputation ranges from the dissertation topic itself, to the corresponding subject area, to related subject areas.

## Conclusion

The medical doctorate is an important instrument for securing scientific knowledge. Despite efforts and revisions in recent years, there are still significant differences between the faculties' regulations with regards to quantitative and qualitative requirements for a doctorate. The most obvious divergences appear in the requirements for publication-based doctorates and participation in GMP, as well as in the formal review process and its underlying criteria.

In order to improve the transparency, comparability and fairness of doctoral performance, it seems desirable and advisable to standardise the doctoral regulations between the faculties.

## Figures and Tables

**Figure 1:**
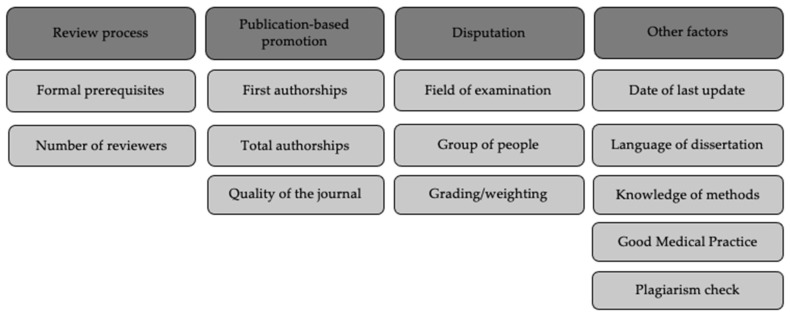
Target criteria.

**Figure 3:**
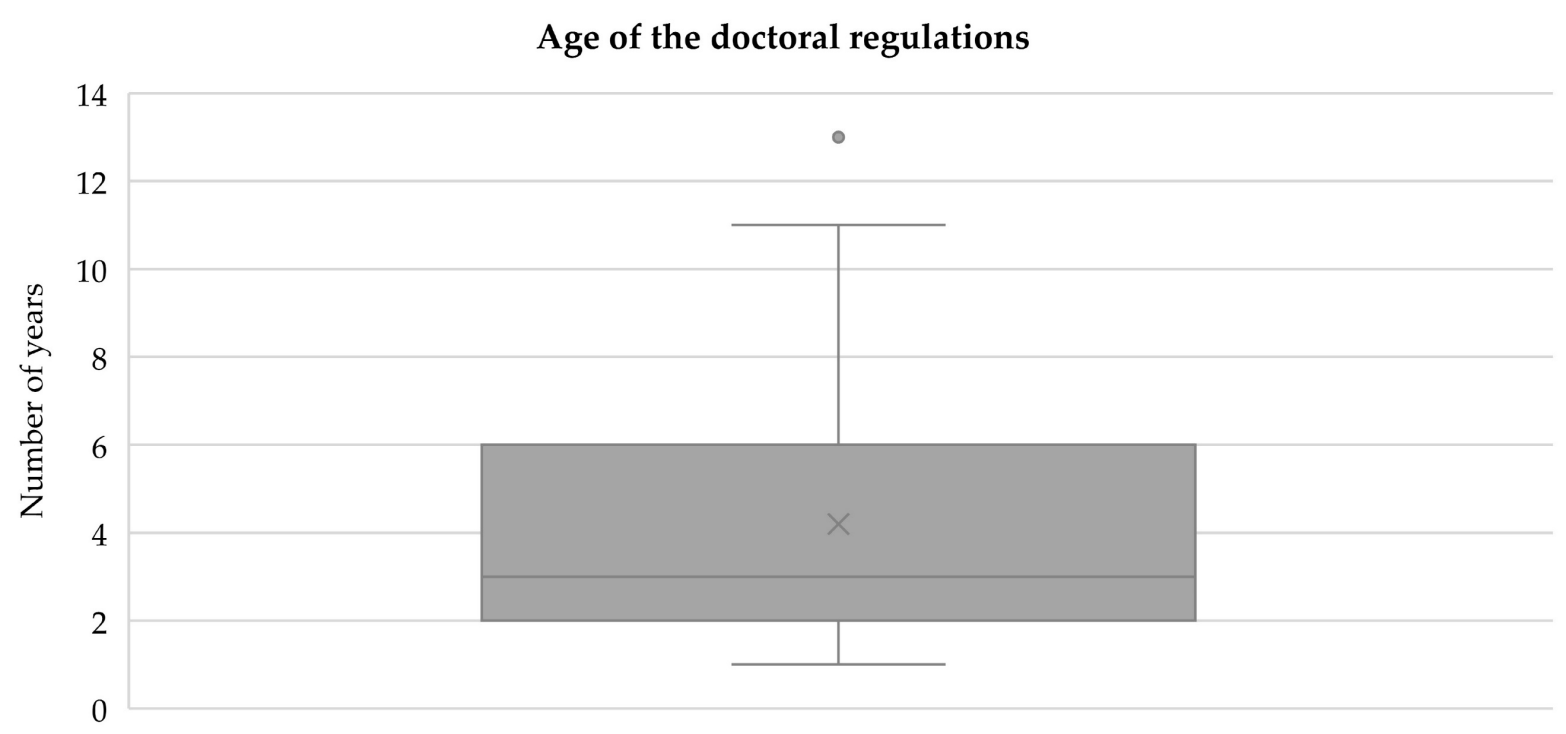
Age of the doctoral regulations.

**Figure 6:**
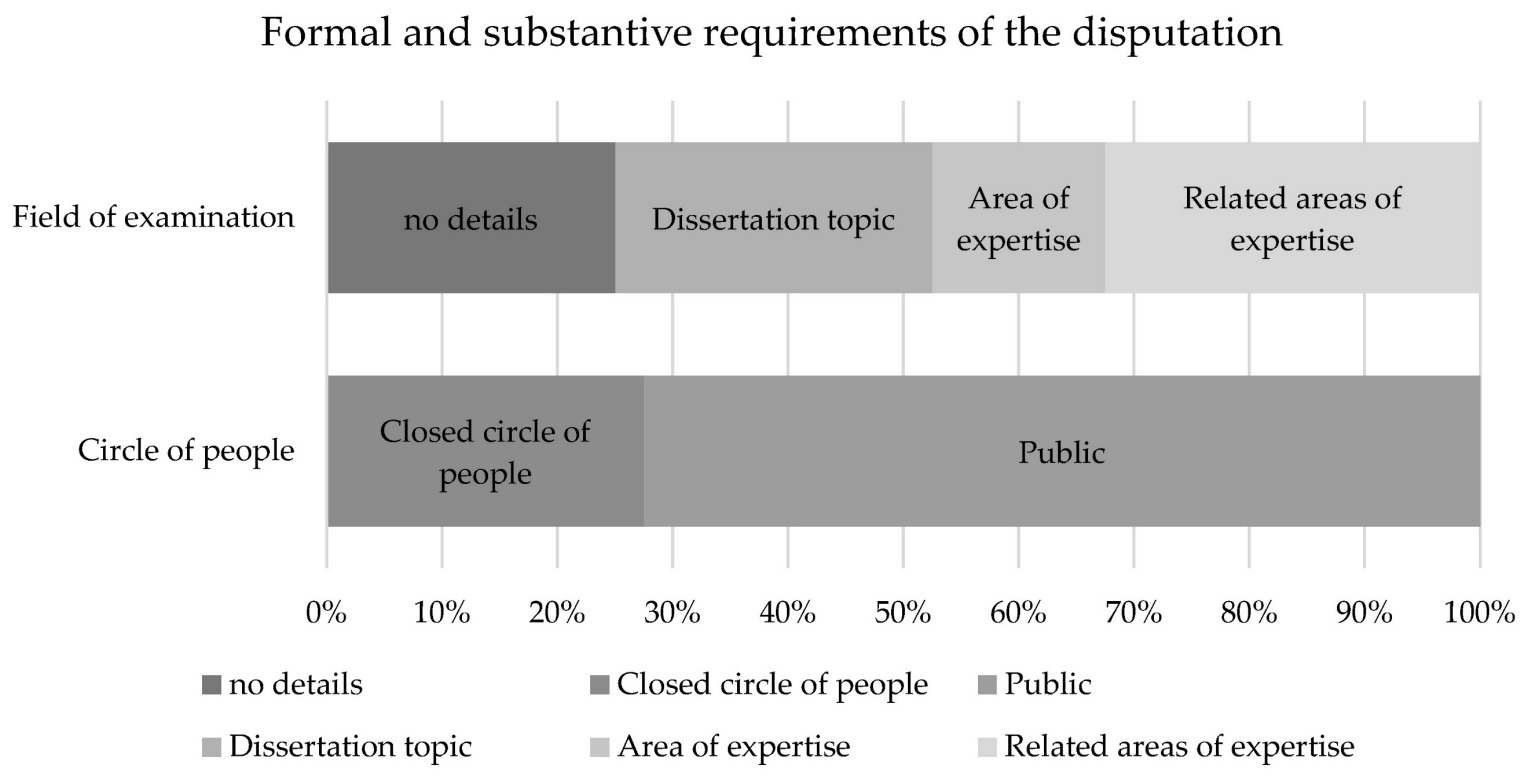
Formal and substantive requirements of the disputation.

## Abbreviations

DFG: Deutsche Forschungsgemeinschaft
GMP: Good Medical Practice
m.c.l.: Magna cum laude
M.D.: Doctor of Medicine
MSTP: Medical scientists programmes
NIH: National Institutes of Health
Ph.D.: Doctor of Philosophy
s.c.l.: Summa cum laude
